# A stress responsive protein contributes to pyomelanin biosynthesis and cell wall stress adaptation in *Aspergillus fumigatus*

**DOI:** 10.3389/fmicb.2026.1934017

**Published:** 2026-09-03

**Authors:** Eduardo Pelegri-Martinez, Uxue Perez-Cuesta, Saioa Cendon-Sanchez, Adela Martin-Vicente, Omaira de la Hera, Arrate Rivas, Rosa M. Alonso, Andoni Ramirez-Garcia, Jarrod R. Fortwendel, Xabier Guruceaga, Aitor Rementeria

**Affiliations:** 1 Department of Immunology, Microbiology and Parasitology of the University of the Basque Country (EHU), Leioa, Spain; 2 Department of Clinical Pharmacy and Translational Science, College of Pharmacy, University of Tennessee Health Science Center (UTHSC), Memphis, TN, United States; 3 FARMARTEM Group, Analytical Chemistry Department, University of the Basque Country (EHU), Leioa, Spain; 4 Institute for Multidisciplinary Research in Applied Biology (IMAB), Public University of Navarre (UPNA), Campus de Arrosadia, Pamplona, Spain

**Keywords:** *Aspergillus fumigatus*, cell wall integrity, homogentisic acid, host-pathogen interactions, phenylalanine/tyrosine catabolism, Pyomelanin, stress response

## Abstract

The biology of the pathogenic fungus *Aspergillus fumigatus* remains largely unexplored, in part due to the large number of hypothetical and uncharacterized proteins. In this study, we focused on the protein encoded by the Afu4g10610 gene, which is consistently up-regulated across multiple stress-related transcriptomic datasets, including both *in vitro* and *in vivo* infection models. Functional characterization through the generation of mutant strains revealed that deletion of Afu4g10610 compromises the response to cell wall stress induced by Congo Red and Calcofluor White, correlating with the downregulation of key cell wall integrity (CWI) pathway sensors (*wsc1* and *midA*). In addition, the mutant exhibits enhanced resistance to osmotic stress, consistent with altered expression of the high-osmolarity glycerol (HOG) pathway effectors *mpkC* and *sakA*. The deletion mutant also showed a moderate reduction in cytotoxicity toward A549 epithelial cells and altered TNF production in RAW 264.7 macrophages, whereas the overexpression strain exhibited a significant decrease in TNF levels. GRAsp analysis predicted this gene to be associated with the phenylalanine/tyrosine catabolic pathway. Accordingly, pyomelanin production and related metabolites were analyzed. Moreover, double deletion mutants ∆*maiA* ∆*10610* and ∆*hmgA* led to a marked reduction in pyomelanin production, accompanied by altered tyrosine consumption and homogentisic acid (HGA) production. Since pyomelanin biosynthesis depends on the conversion of HGA into benzoquinone acetate (BQA), a step traditionally considered spontaneous, we investigated the potential interaction between Afu4g10610 and HGA. Molecular docking analysis supported the binding of HGA at the predicted dimer interface of the protein and suggested potential binding of FADH₂ to the protein, as an electron donor. Together, these findings identify Afu4g10610 as a stress-associated protein that contributes to cell wall and osmotic stress adaptation and suggest a potential contribution to HGA-to-BQA conversion during pyomelanin biosynthesis. More broadly, our results support the possible involvement of an enzymatic component in a step previously considered spontaneous in *A. fumigatus*.

## 1 Introduction

*Aspergillus fumigatus* is the most common pathogenic species within the genus *Aspergillus* (Latgé and Chamilos, 2019) and has been classified by the World Health Organization as a fungal pathogen within the Critical Priority Group (World Health Organization, 2022). This fungus frequently causes invasive infections with high mortality rates (Arastehfar et al., 2021; Brown et al., 2012; Lamoth and Calandra, 2022). Furthermore, azole-resistant *A. fumigatus* strains have increased in recent years, underscoring the need to study fungal virulence and identify new diagnostic and therapeutic targets to address this public health concern (Denning, 2024). The virulence of this fungus is driven by its ability to adapt to hostile environments, enabling tissue invasion, immune evasion, and antifungal resistance (Paulussen et al., 2017; Slesiona et al., 2012; Van De Veerdonk et al., 2025). Despite these advances, significant gaps remain in our understanding of *A. fumigatus* infection. Of the approximately 15,000 genes in its genome, around 60% remain poorly studied and encode proteins with unknown, hypothetical, or unclassified functions (Barber et al., 2021; Latgé and Chamilos, 2019; Lofgren et al., 2022). Accordingly, our research group has focused on identifying infection-relevant *A. fumigatus* genes and characterizing their functions through mutant strain analysis. Specifically, by comparing transcriptomic data from different *in vivo* and *in vitro* infection models, we detected 13 genes that were consistently up-regulated across all three infection conditions, including different cell lines (murine macrophages and human lung epithelial cells), as well as in a previously published *in vivo* infection model (Guruceaga et al., 2018, 2024).

Notably, this set of 13 up-regulated genes comprises components of the phenylalanine/tyrosine catabolic pathway (*hppD* and *maiA*). These genes belong to a cluster on chromosome 2 that includes six genes (*hppD*, *hmgX*, *hmgA*, *fahA*, *maiA*, and *hmgR*), that are coordinately transcribed (Schmaler-Ripcke et al., 2009). This pathway is associated with the production of the secondary metabolite, pyomelanin, an extracellular dark brown pigment secreted during hyphal growth (Heinekamp et al., 2013; Schmaler-Ripcke et al., 2009). Within this pathway, pyomelanin formation is preceded by the conversion of a downstream intermediate, homogentisic acid (HGA), into benzoquinone acetate (BQA). This step involves monooxygenation of the aromatic ring and constitutes a key event in pyomelanin biosynthesis. This reaction has traditionally been considered a spontaneous process since no specific enzyme has been identified to catalyze this step in filamentous fungi. Subsequently, BQA undergoes auto-polymerization, leading to pigment formation. Pyomelanin has been linked to stress resistance, immune evasion, and fungal survival, highlighting its potential relevance in pathogenesis, although its precise role in *A. fumigatus* biology and host responses remains unclear (Guruceaga et al., 2024; Heinekamp et al., 2013; Perez-Cuesta et al., 2020). Our research group has previously studied one of the 13 genes detected as overexpressed in the infection models linked to this pathway, the *maiA* gene (Afu2g04240). This gene encodes a maleylacetoacetate isomerase and has been linked to both virulence and fungal cell wall homeostasis (Guruceaga et al., 2024). Among the 13 overexpressed genes, several genes remain poorly characterized or encode hypothetical proteins, including Afu4g10610, Afu6g00430, Afu6g02210, Afu1g04160, Afu5g02320, and Afu6g12250.

In this study, we focused on Afu4g10610, a gene encoding a protein of unknown function that emerged as a stress-associated candidate in multiple transcriptomic datasets. Based on GRAsp analysis (Gene Regulation of *Aspergillus fumigatus*) (Carriel et al., 2026; Seo et al., 2025) which predicted its involvement in the phenylalanine/tyrosine catabolic pathway, we investigated the function of the Afu4g10610 through the generation of mutant strains, assessing its role in stress adaptation, pyomelanin biosynthesis, and host-interaction responses.

## 2 Materials and methods

### 2.1 *Aspergillus fumigatus* strains and growth conditions

The *A. fumigatus* Af293 strain was used as the wild type (WT) genetic background to develop the mutant strains generated in this study. All the strains were grown in Glucose Minimal Medium (GMM, 6 g NaNO_3_, 0.52 g KCl, 0.52 g MgSO_4_∙7H_2_O, 1.52 g KH_2_PO_4_, 1 mL trace elements [2.2 g ZnSO_4_∙7H_2_O, 1.1 g H_3_BO_3_, 0.5 g MnCl_2_∙4H_2_O, 0.5 g FeSO_4_∙7H_2_O, 0.16 g CoCl_2_∙6H_2_O, 0.16 g CuSO_4_∙5H_2_O, 0.11 g (NH_4_)_6_Mo_7_O_24_∙4H_2_O, 5 g EDTA in 100 mL distilled H_2_O], 10 g glucose, 15 g agar, pH 6.5, in 1 L distilled H_2_O) at 37 °C. Conidia were harvested and washed twice with saline-Tween solution (SS-T; 0.9% NaCl and 0.02% Tween 20), and their concentration was determined using a Bürker counting chamber.

### 2.2 Cell lines

The murine macrophage cell line RAW 264.7 and the human lung epithelial cell line A549, both obtained from the American Type Culture Collection (ATCC, Manassas, VA, USA), were used in this study. Both cell lines were grown in RPMI 1640 supplemented with 10% of heat-inactivated fetal bovine serum (VWR, Radnor, PA, USA), L-glutamine (2 mM) (VWR), penicillin (10,000 U/mL), streptomycin (10 mg/mL) and amphotericin B (25 μg/mL) (Thermo Fisher Scientific, Waltham, MA, USA) at 37 °C, 5% CO_2_ and 95% humidity. Cells were passaged upon reaching 80–90% confluence. Cell concentration and viability were assessed by staining an aliquot with 0.4% Trypan Blue Solution (Merck, Darmstadt, Germany) and counting in a Bürker counting chamber. Only cultures with viability exceeding 95% were used for subsequent assays.

### 2.3 Genetic manipulations

To carry out deletion strains, CRISPR/Cas9-mediated gene targeting was performed in the Af293 background as previously described (Al Abdallah et al., 2017). In brief, the Δ*10610* deletion strain was generated by replacing the entire Afu4g10610 coding sequence with a hygromycin resistance cassette (HygR) (Supplementary Figure S1A). The repair template was designed with 40 bp microhomology regions flanking the target locus, corresponding to sequences adjacent to selected protospacer adjacent motifs (PAMs). Two PAM sites located upstream and downstream of the gene were selected, and potential off-target effects were evaluated during guide RNA design. Guide RNAs (gRNAs) and ribonucleoprotein (RNP) complexes were assembled *in vitro* using commercially available tracrRNA and Cas9 nuclease (IDT, Integrated DNA Technologies Inc., USA) (Al Abdallah et al., 2017; Souza et al., 2021). Complementation of the Δ*10610* mutant (∆*10610*^comp^) was carried out by CRISPR/Cas9-mediated repair using a phleomycin resistance cassette (PhleoR), amplified from the plasmid pAGRP (Fortwendel et al., 2012). The Afu4g10610 gene was amplified from genomic DNA and fused to the phleomycin resistance cassette by fusion PCR to generate the donor cassette (Supplementary Figure S1B). For the generation of the overexpression strain (OE:*10610*), the native promoter of the Afu4g10610 gene was replaced with the strong *hspA* promoter, as previously described (Paul et al., 2012; Xie et al., 2024). A single PAM site located upstream of the coding region was selected to direct the insertion. The repair template was constructed by amplifying the PhleoR fused to the *hspA* promoter, enabling constitutive expression of Afu4g10610 (Supplementary Figure S1C). Finally, double deletion mutants were generated using two different selectable markers, each corresponding to one of the deleted loci (Supplementary Figure S1D). All primers and crRNAs used are listed in Supplementary Table S1.

For transformation, 1–5 × 10^5^ protoplasts were combined with the Cas9-RNP complex (1.2 μM gRNA and 500 ng Cas9), the repair template (~500 ng), 60% polyethylene glycol 3,350 (PEG), and 11 μL of STC buffer (1.2 M sorbitol, 7.55 mM CaCl_2_·H_2_O, 10 mM Tris–HCl, pH 7.5), and the mixture was incubated on ice. After 50 min, an additional 57 μL of PEG-CaCl_2_ was added, followed by incubation at room temperature for 20 min. The mixture was then diluted with 113 mL of STC buffer to reduce viscosity, and the protoplasts were plated onto sorbitol minimal medium (SMM) agar plates, consisting of GMM supplemented with 218.6 g/L sorbitol. After overnight incubation at room temperature, selection was carried out by overlaying the plates with top agar (SMM supplemented with 7 g/L agar) containing hygromycin B (150 μg/mL) or phleomycin (125 μg/mL). Plates were incubated at 37 °C for 3 days for hygromycin selection or at 30 °C for 5 days for phleomycin selection. Individual colonies were subsequently transferred to fresh selective media, and putative transformants were purified by single-spore isolation and verified by PCR and sequencing.

### 2.4 Bioinformatic analysis

Protein structure prediction was performed using AlphaFold (https://alphafold.ebi.ac.uk/) (Jumper et al., 2021) and AlphaFold3 (https://alphafoldserver.com/welcome) (Abramson et al., 2024). Model confidence was assessed using the predicted local distance difference test (pLDDT), which provides a per-residue confidence score ranging from 0 to 100, with higher values indicating greater reliability in local structural predictions. In addition, the predicted aligned error (PAE) was used to evaluate the relative positioning of residues or domains within the model, where lower values indicate higher confidence in the global arrangement of the structure. Functional predictions and pathway associations were assessed using the bioinformatics platform GRAsp (https://grasp.wid.wisc.edu) (Carriel et al., 2026; Seo et al., 2025). Molecular docking analyses were carried out using SwissDock (https://swissdock.ch) (Bugnon et al., 2024; Grosdidier et al., 2011) to evaluate potential interactions between the protein and candidate ligands. The methods used were Attracting Cavities (Röhrig et al., 2023; Zoete et al., 2016) and AutoDock Vina (Eberhardt et al., 2021; Trott and Olson, 2010). Visualization and analysis of predicted protein structures and docking poses were performed using UCSF Chimera 1.15 (Pettersen et al., 2004).

### 2.5 Stress resistance and antifungal susceptibility assays

Mutant strains were screened for stress sensitivity by monitoring growth in the presence of different stress-inducing agents. Congo red (CR; Sigma-Aldrich, St. Louis, MO, USA) and calcofluor white (CFW; MP Biomedicals, Germany) were used at a final concentration of 60 μg/mL. Osmotic stress conditions were assessed using sorbitol minimal medium (SMM). Additional osmotic stress conditions were evaluated by supplementing media with either 1.2 M KCl or 1.2 M NaCl as indicated. To evaluate the ability of the strains to utilize phenylalanine (Phe) or tyrosine (Tyr) as carbon and/or nitrogen sources, GMM and minimal medium (MM; GMM without glucose) plates were supplemented with 10 mM Phe or 10 mM Tyr. In addition, nitrogen-limited conditions were assessed using media lacking NaNO₃, including MM without NaNO₃ supplemented with Tyr as the sole carbon and nitrogen source. Spot dilution assays were performed as previously described (Martin-Vicente et al., 2019). Briefly, 5 μL drops containing 1 × 10^4^, 10^3^, and 10^2^ conidia were inoculated onto GMM plates supplemented with the corresponding stress agents and incubated at 37 °C for 72 h. For radial growth measurements, 10 μL drops containing 1 × 10^5^ conidia were inoculated at the center of 60 mm Petri dishes containing the indicated media. Plates were incubated at 37 °C, and colony diameters were measured along two perpendicular axes at 24, 48, and 72 h. Antifungal susceptibility was assessed using two complementary approaches. For the gradient diffusion assay, 1 × 10^6^ conidia were spread onto agar plates, and Etest® strips (bioMérieux, Marcy-l’Étoile, France) for caspofungin and micafungin were placed 15 min after inoculation. Plates were incubated at 37 °C and read after 48 h. The minimum inhibitory concentration (MIC) was defined as the lowest drug concentration at which the border of the elliptical inhibition zone intersected the scale on the strip. For plate-based susceptibility assays, GMM plates were supplemented with two-fold serial dilutions of caspofungin or nikkomycin Z (Merck, Darmstadt, Germany), starting from 8 μg/mL. Each condition was inoculated with a 5 μL drop containing 10^4^ conidia and incubated at 37 °C for 72 h. GMM plates without supplements were used as growth controls, and all experiments were performed in at least biological triplicate.

### 2.6 RNA extraction and RT-qPCR expression analysis

Specific *A. fumigatus* primers were designed using Primer3 (https://primer3.ut.ee/) and the IDT PrimerQuest™ tool (Integrated DNA Technologies Inc., USA; https://.eu.idtdna.com/site). Primer specificity was evaluated using Primer-BLAST (https://.ncbi.nlm.nih.gov/tools/primer-blast/) to avoid non-specific amplification, and potential primer-dimer and self-dimer formation was assessed using the Multiple Primer Analyzer tool (Thermo Fisher Scientific; https://.thermofisher.com). For gene expression analysis, 1 × 10^6^ conidia/mL were inoculated in GMM and incubated at 37 °C for 16 h under constant agitation (250 rpm), as previously described (Martin-Vicente et al., 2020). Mycelia were harvested, washed twice with distilled water, and ground in liquid nitrogen. Total RNA was extracted using the NZY Total RNA Isolation kit (NZYtech, Lisbon, Portugal), and RNA concentration and purity were assessed spectrophotometrically using a NanoDrop instrument (ThermoFisher). Genomic DNA contamination was removed by treating 5 μg of total RNA with Turbo DNase (Invitrogen). cDNA synthesis was performed using the NZY First-Strand cDNA Synthesis Kit (NZYtech), and quantitative real-time PCR was carried out using NZYSupreme qPCR Green Master Mix (NZYtech). Relative gene expression levels were calculated using the 2^-∆∆Ct^ method (Livak and Schmittgen, 2001) with the *tubA* gene (Afu1g10910), encoding *β*-tubulin, used as housekeeping gene. The following genes were analyzed: those involved in aromatic amino acid metabolism (*hppD, hmgA, idoC*); cell wall biosynthesis and remodeling (*fks1, cwh41, chsA, chsC*); the cell wall integrity (CWI) pathway (*wsc1, midA, rho1, mpkA, rlmA*); the high osmolarity glycerol (HOG) pathway (*sakA, mpkC*); and developmental and morphogenetic regulation (*brlA, wetA, rasB*), as well as key regulatory genes such as *crzA, laeA,* and *srbA*. Gene selection was based on their involvement in relevant metabolic and signaling pathways. All primers are listed in Supplementary Table S1. Each experiment was performed independently in biological triplicate, with three technical replicates per condition.

### 2.7 Determination of pyomelanin production

Pyomelanin production was measured following previously described procedures (Guruceaga et al., 2024; Schmaler-Ripcke et al., 2009). Briefly, GMM broth (150 mL) was inoculated with 1 × 10^7^ conidia of each indicated *A. fumigatus* strain. After 20 h of pre-incubation, Tyr was added to a final concentration of 10 mM, marking the start point (time 0) for subsequent measurements. Aliquots of 500 μL were collected at 24, 48, and 72 h. Pigment formation was assessed by measuring the absorbance at 405 nm of alkalinized supernatants obtained by adding 20 μL of 5 M NaOH per mL of sample, followed by centrifugation at 16,000 g for 2 min. Three independent biological replicates were performed on different days.

### 2.8 HPLC-PDA quantification of tyrosine and homogentisic acid

Tyrosine and HGA concentrations were determined by High-Performance Liquid Chromatography coupled to a Photodiode Array detector (HPLC-PDA). For sample preparation, 250 μL of each sample were diluted 1:2 in ultrapure Milli-Q water containing 0.1% trifluoroacetic acid (TFA; Scharlab, Barcelona, Spain). Samples were vortex-mixed, filtered through 0.22 μm filters, and transferred into 300 μL inserts for 2 mL vials prior to injection into the HPLC system. Chromatographic analysis was performed using a 2,695 HPLC system coupled to a 996-photodiode array detector (Waters, Milford, MA, USA). Separation was achieved using a C18 Zorbax Eclipse XDB column (15 cm × 4.6 mm, 5 μm; Agilent Technologies, Santa Clara, CA, USA) equipped with a C18 pre-column (4 × 3.0 mm; Sigma-Aldrich). The mobile phases consisted of ultrapure Milli-Q water with 0.1% TFA (A) and HPLC-grade acetonitrile (B; Scharlab). Chromatographic peaks were acquired and integrated using Empower 3 software (Waters). The specific HPLC-PDA conditions used for tyrosine and HGA analysis are given in Supplementary Table S2. The concentrations of the analytes were obtained by the interpolation of the chromatographic peak areas in the calibration curves, constructed with standard solutions of L-tyrosine and HGA. The Area Under the Curve (AUC) was calculated from the tyrosine and HGA concentration time-course data using the trapezoidal integration method (Tai, 1994).

### 2.9 Infection assays

Cytotoxicity was assessed by measuring lactate dehydrogenase (LDH) release in A549 cell line using the LDH-Cytox™ Assay Kit (BioLegend, San Diego, USA), following adapted manufacturer’s instructions. A549 epithelial cells were infected with each *A. fumigatus* strain at a multiplicity of infection (MOI) of 1 for 16 h. Briefly, 100 μL of supernatant from each condition was transferred to an optically clear 96-well plate in triplicate and mixed with 100 μL of working solution (1:1). Plates were protected from light and incubated at room temperature for 30 min. The reaction was stopped by adding 50 μL of stop solution, and absorbance was measured at 490 nm. Uninfected cells were used as negative controls, while maximum LDH release was determined by treating cells with lysis buffer. A fungus-only control was included and showed no detectable signal.

Macrophage-mediated conidial killing was assessed as previously described with minor modifications (Goldmann et al., 2023; Wei et al., 2025). Briefly, 1 × 10^6^ RAW 264.7 macrophages were seeded in 2 mL of complete RPMI medium in 6-well plates and incubated for 24 h to allow adherence and confluence. Cells were then infected with conidia from wild-type and mutant strains at MOI of 2. After 6 h of incubation at 37 °C with 5% CO₂, samples were collected and lysed in Milli-Q water containing 0.1% (v/v) Tween 20. Serial dilutions were plated on Sabouraud dextrose agar (VWR, Radnor, PA, USA) and incubated for 36 h at 37 °C. Colony-forming units (CFUs) were counted to determine the number of surviving conidia. Macrophage conidial killing (%) was calculated as: (conidia added - conidia recovered) / conidia added. Experiments were performed independently three times.

Phagocytosis assays was performed using RAW 264.7 macrophages seeded at a density of 2 × 10^5^ cells/mL in 500 μL of complete RPMI 1640 medium (supplemented with 10% heat-inactivated FBS, 2 mM L-glutamine, 100 U/mL penicillin, and 0.1 mg/mL streptomycin) onto 12 mm coverslips placed in 24-well plates. Following overnight incubation to allow cell adherence, macrophages were co-incubated with *A. fumigatus* conidia at a MOI = 10 in 500 μL of infection medium (RPMI 1640 supplemented with 10% heat-inactivated FBS and 2 mM L-glutamine, without antibiotics). In parallel, equivalent conidial suspensions were incubated in the absence of macrophages as controls. At 4, 6, 8, and 10 h post-infection, coverslips were transferred to a new plate containing cold PBS to stop phagocytosis. Samples were then analyzed using an inverted microscope (Eclipse TE2000-U, Nikon, Tokyo, Japan) to determine phagocytosis rates. Phagocytosis percentage was calculated as ([phagocytic macrophages/total macrophages] × 100). For each assay, three biological replicates were analyzed, counting at least 100 macrophages across five fields of view per well. TNF release by macrophages was quantified by enzyme-linked immunosorbent assay (ELISA) using the Mouse TNF-*α* ELISA MAX™ Deluxe Set (BioLegend, Cat. N°430,904), following the manufacturer instructions.

### 2.10 Scanning electron microscopy

For cell wall surface analysis, 5 × 10^5^ conidia/mL from Af293 and Δ*10610* were seeded onto coverslips submerged in GMM broth. Samples were incubated at 37 °C for 16 h. Coverslips were then fixed and sequentially dehydrated through increasing ethanol concentrations followed by hexamethyldisilazane treatment. Finally, samples were sputter-coated with gold under an argon atmosphere and observed using a scanning electron microscope (Hitachi S-4800) at the Research General Services (SGIker) of the University of the Basque Country (EHU).

### 2.11 Statistics

Statistical analysis was performed using GraphPad Prism v. 8.0.2 (GraphPad Software Inc., San Diego, CA, USA). For comparisons between two groups, statistical significance was assessed using a two-tailed unpaired Student *t*-test. For multiple group comparisons and experiments involving two independent variables, a two-way analysis of variance (ANOVA) followed by Tukey’s multiple comparisons test was applied. Data are presented as mean ± standard deviation (SD). Statistical significance was defined as *p < 0.05, **p < 0.01, ***p < 0.001, and ****p < 0.0001.

## 3 Results

### 3.1 Predicted *α*/*β*-barrel homodimer structure of the Afu4g10610 protein and its association with aromatic amino acid metabolism

**Figure 1:**
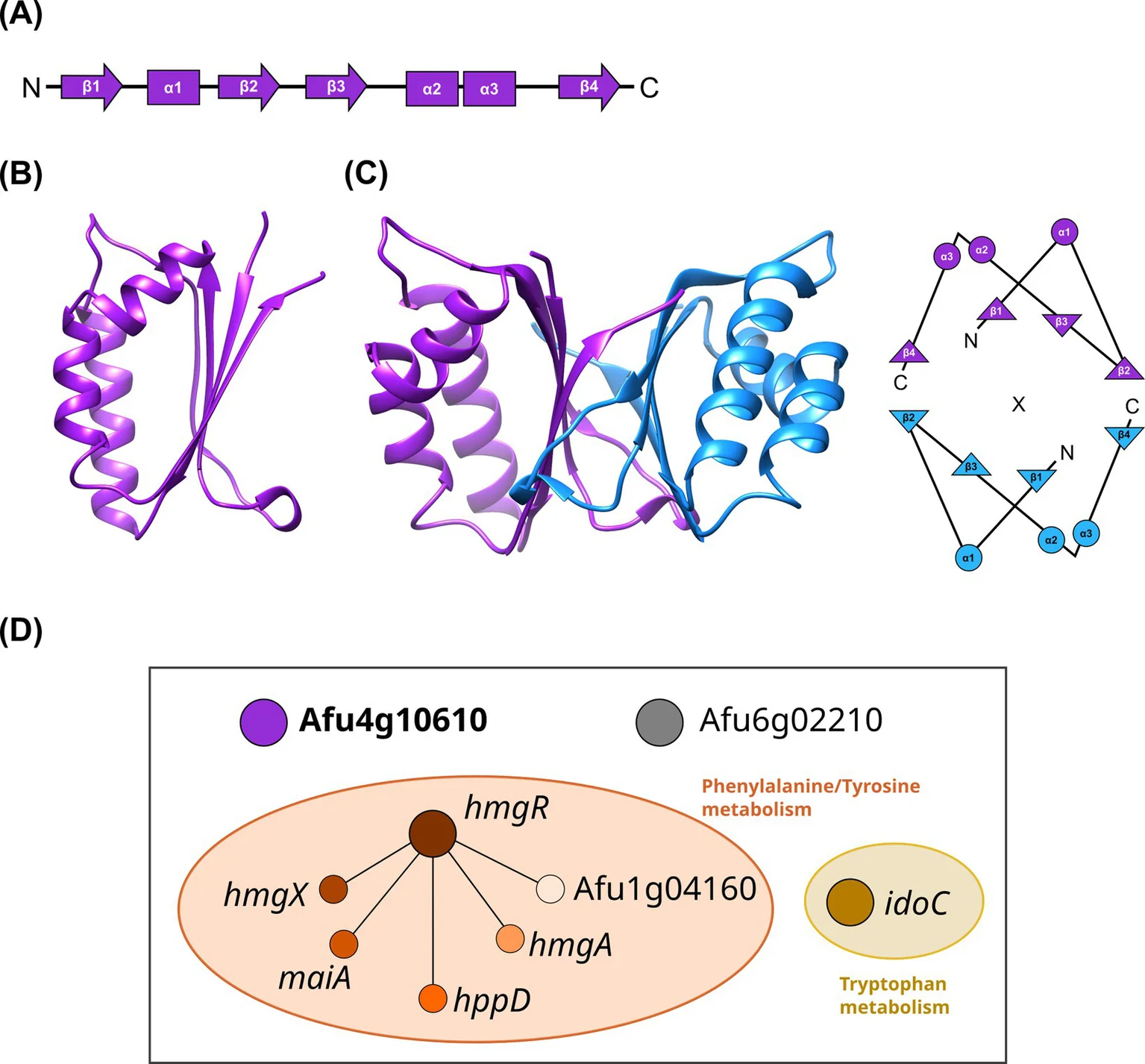
Predicted model of the Afu4g10610 protein: **(A)** Schematic representation of the protein structure, N and C indicate the amino- and carboxy-terminal ends of the protein, respectively. **(B)** Three-dimensional model of the monomer. **(C)** Dimer model showing the two facing monomers. The X marks the central pore. The models were predicted using AlphaFold and represented with Chimera 1.15. **(D)** GRAsp output (Module: 5208) showing predicted relationships between Afu4g10610 and other genes involved in aromatic amino acid metabolism. Colored clusters highlight the phenylalanine/tyrosine metabolism (orange), tryptophan metabolism (*idoC*, yellow), Afu4g10610 gene (purple), and Afu6g02210 gene (gray) as indicated in the figure. Edges represent predicted regulatory interactions based on gene co-expression and genomic context.

The Afu4g10610 protein (UniProt: Q4WPV7) consists of 104 amino acids that are predicted to fold into three α-helices and four β-strands (Figure 1A), and belongs to the dimeric α/β barrel (Dabb) protein family. AlphaFold prediction of Afu4g10610 monomeric structure (Figure 1B) achieved a high per-residue confidence, with a pLDDT score of 97.68, indicating reliable atomic positioning throughout the structure. This model also exhibited a low PAE, suggesting accurate relative positioning of residue pairs (Supplementary Figure S2A).

The α/β-barrel fold typically forms homodimeric assemblies, although heterodimeric conformations have also been reported. Using AlphaFold3 multimeric prediction tool, the Afu4g10610 monomer was predicted to assemble into a homodimeric α/β-barrel architecture. In this arrangement, the β-strands of each subunit adopt an antiparallel orientation, and the outer surfaces of each β-sheet form a barrel structure surrounding a central pore (Figure 1C). This predicted dimer model displayed high confidence, with pLDDT scores above 90 and low PAE values (Supplementary Figure S2B). Additionally, the dimer prediction yielded a pTM (predicted Template Modeling) score of 0.94 and an ipTM (inter-protein TM-score) of 0.93, indicating that the overall folding of the complex is reliable and the relative positions of the subunits are well-predicted, as ipTM values higher than 0.8 represent confident high-quality predictions. The predicted interface shows high-confidence metrics, although experimental validation is required to confirm dimerization *in vivo*.

Furthermore, using the bioinformatic tool GRAsp, we identified potential relationships between the Afu4g10610 gene and other enzyme-coding genes involved in aromatic amino acid degradation. These include the complete Phe/Tyr degradation cluster, which is closely linked to the production of the secondary metabolite pyomelanin, as well as the monooxygenase encoded by Afu6g02210 and the indoleamine 2,3-dioxygenase encoded by Afu7g02010 (*idoC*). Both genes, together with several members of the Phe/Tyr degradation cluster (including *maiA* and *hppD*), were previously reported to be upregulated in infection models (see Introduction) (Figure 1D).

### 3.2 Role of Afu4g10610 protein in cell wall integrity and osmotic stress response

To elucidate the functional role of the Afu4g10610 protein and its relationship with these metabolic pathways, several mutant strains were generated using highly efficient CRISPR-Cas9-based genetic engineering. The deletion strain (Δ*10610*) was constructed by replacing the gene locus with the HygR marker (Supplementary Figure S1A). The complemented strain (∆*10610*^comp^) was created by restoring the native gene followed by the PhleoR marker at the original locus (Supplementary Figure S1B). The overexpression strain (OE:*10610*) was generated by introducing the *hspA* promoter upstream of the Afu4g10610 gene, with the PhleoR gene oriented in the opposite direction (Supplementary Figure S1C). Additionally, to explore interactions with the key enzymes of the Phe/Tyr degradation pathway, we used the Δ*maiA* strain, previously generated in our laboratory, together with the Δ*maiA* Δ*10610* (Supplementary Figure S1D), and Δ*10610* Δ*hmgA* strains, allowing us to assess the combinatorial effects of these deletions on Phe/Tyr catabolism.

**Figure 2:**
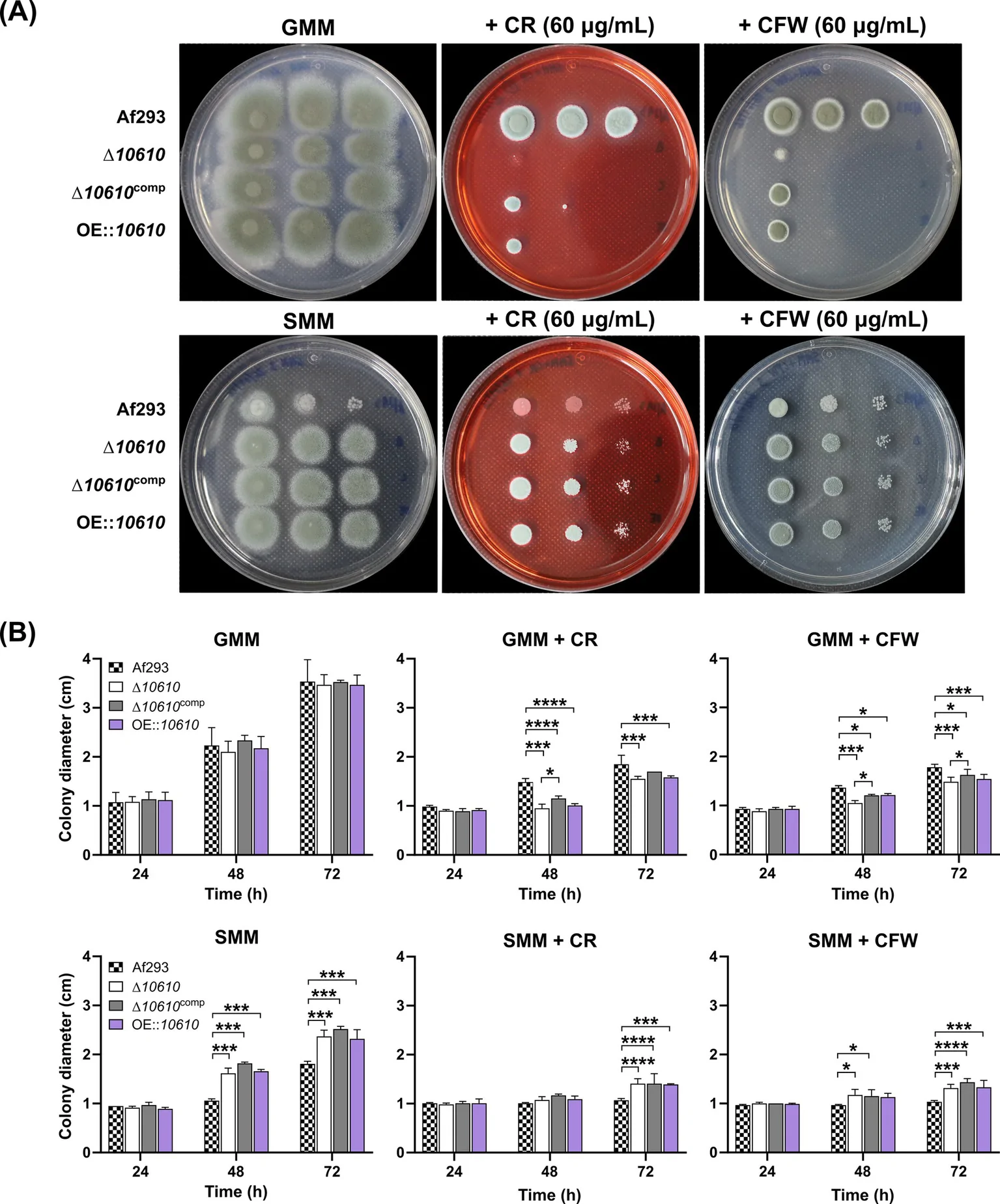
Phenotypic characterization of Af293, ∆*10610*, ∆*10610*^comp^, and OE:*10610* strains. **(A)** Phenotypic analysis by spot dilution assays under cell wall and osmotic stress conditions. The strains were spotted (10^4^, 10^3^, and 10^2^ conidia) on GMM, GMM supplemented with 60 μg/mL Congo Red (CR) or Calcofluor White (CFW), and SMM, and incubated at 37 °C for 72 h, after which pictures were taken. **(B)** Colony diameters were measured after spotting 10^5^ conidia under the same conditions and recorded at 24, 48, and 72 h. Data were analyzed by one-way ANOVA with Tukey’s multiple comparisons test (*n* = 3 independent experiments; mean ± *SD*). Statistical significance is indicated as follows: *p < 0.05, ***p < 0.001, ****p < 0.0001.

We evaluated the growth of the Δ*10610*, Δ*10610*^comp^, and OE:*10610* mutants under different conditions to assess the contribution of this gene to stress adaptation. No significant differences in growth were observed among the strains under non-stress conditions (GMM) (Figure 2A). However, cell wall and osmotic stress conditions led to clear phenotypic differences. Under cell wall stress induced by CR and CFW, the Δ*10610* mutant exhibited a significant reduction in colony diameter compared to Af293 strain, indicating increased sensitivity to these stressors (Figure 2A). This phenotype was clearly visible in plate assays and confirmed by quantitative colony diameter measurements (Figure 2B). The complemented strain (∆*10610*^comp^) and the overexpression strain (OE:*10610*) partially restored the wild-type phenotype.

Despite the increased sensitivity to CR and CFW, the Δ*10610* mutant did not exhibit increased susceptibility to antifungal agents targeting the fungal cell wall, including the chitin synthase inhibitor nikkomycin Z (Supplementary Figure S3A) and the echinocandins caspofungin and micafungin, as assessed by plate assays and E-test (Supplementary Figures S3B,C). Likewise, susceptibility to the azoles voriconazole and posaconazole, evaluated by Etest®, was similar to the wild-type strain (Supplementary Figure S3C). Furthermore, no differences were observed under additional stress conditions, including heat stress (48 °C), heat stress with osmotic stabilization (48 °C, GMM supplemented with 1.2 M sorbitol), caffeine-induced stress (5 mM), or alkaline stress (pH 9) (Supplementary Figure S3G). Finally, scanning electron microscopy (SEM) analysis revealed no major structural differences in the cell wall architecture of the Δ*10610* mutant compared to the WT strain (Supplementary Figure S3D).

On the other hand, the Δ*10610,* ∆*10610*^comp^, and OE:*10610* mutants exhibited significantly enhanced growth under hyperosmotic conditions, including SMM (Figure 2A) and GMM supplemented with KCl or NaCl (Supplementary Figure S3E), indicating enhanced tolerance to osmotic stress. Notably, sorbitol supplementation also improved tolerance to cell wall stress induced by CR or CFW (Figure 2B).

**Figure 3:**
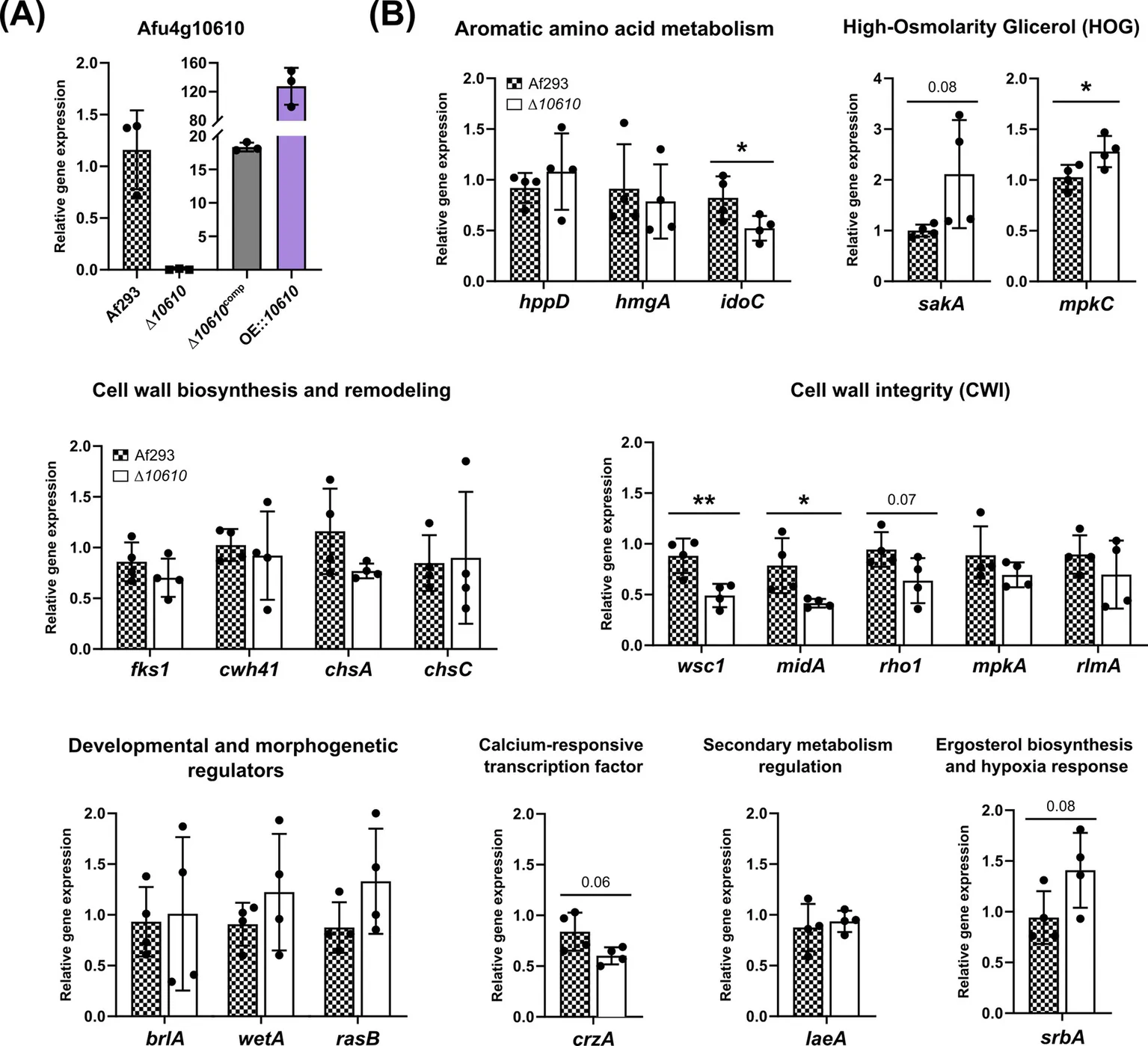
RT-qPCR gene expression analysis. **(A)** Afu4g10610 gene expression **(B)** Expression of selected genes involved in aromatic amino acid metabolism, HOG and CWI pathways, cell wall biosynthesis and remodeling, developmental and morphogenetic regulation, a calcium-responsive transcription factor, secondary metabolism regulation, and ergosterol biosynthesis and hypoxia response. Gene expression levels in Af293 and ∆*10610* were calculated using the 2^-∆∆Ct^ method and normalized to the housekeeping gene *tubA*. Statistical significance was assessed using unpaired two-tailed Student’s *t*-tests performed independently for each gene. Data represent mean ± SD of four biological replicates. *p < 0.05, **p < 0.01.

To investigate the molecular basis underlying these phenotypes, we used RT-qPCR to analyze the expression of the Afu4g10610 gene and several genes involved in *A. fumigatus* metabolism (Figure 3). Notably, the complemented strain displayed higher Afu4g10610 expression levels than Af293 strain, although lower than those observed in the OE:*10610* strain, which may explain their similar phenotypic behavior and the altered response relative to wild-type strain (Figure 3A). No differences in gene expression were detected between the WT strain and the deletion mutant in genes such as, *fks1, cwh41, chsA*, or *chsC*, related with cell wall biosynthesis and remodeling (Figure 3B). However, when analyzing gene expression within the CWI pathway, we detected a statistically significant downregulation of the sensor genes *wsc1* and *midA*. A similar trend toward reduced expression, although not statistically significant, was observed for *rho1*, *mpkA*, and the transcription factor-encoding gene *rlmA*, indicating a partial impairment of the CWI signaling pathway in Δ*10610* strain. This expression pattern correlates with the sensitivity observed under cell wall stress induced by CR and CFW, which directly perturb cell wall integrity, while no increased susceptibility to antifungal agents targeting cell wall biosynthesis was observed.

Regarding the HOG pathway, which mediates the response to osmotic stress, we assessed the expression of *mpkC* and *sakA*, which encode the main effectors of the pathway. The *mpkC* gene was significantly up-regulated in the Δ*10610* mutant. A similar trend was observed for *sakA* gene, although it was not statistically significant. These findings are consistent with the enhanced osmotic stress response of the mutants and with the osmotic stabilization observed under cell wall stress conditions. Among the other genes studied, a significant decrease in expression was observed for the *idoC* gene, encoding an enzyme involved in tryptophan metabolism. A similar but non-significant trend was observed for *crzA*, a calcium- and calcineurin-dependent transcription factor (TF), and *srbA*, which encodes the sterol regulatory element-binding protein that plays a key role in ergosterol biosynthesis and the hypoxia response. Finally, no significant differences were detected in genes regulating key processes such as development and morphogenesis or secondary metabolism in this fungus.

Overall, these results highlight the inability of the Δ*10610* mutant to respond to cell wall stress and the increased resistance to osmotic stress observed, suggesting a specific role for Afu4g10610 protein in modulating these two stress responses, potentially with effects on other central pathways involved in *A. fumigatus* metabolism and adaptation.

### 3.3 Afu4g10610 protein regulates pyomelanin biosynthesis

**Figure 4:**
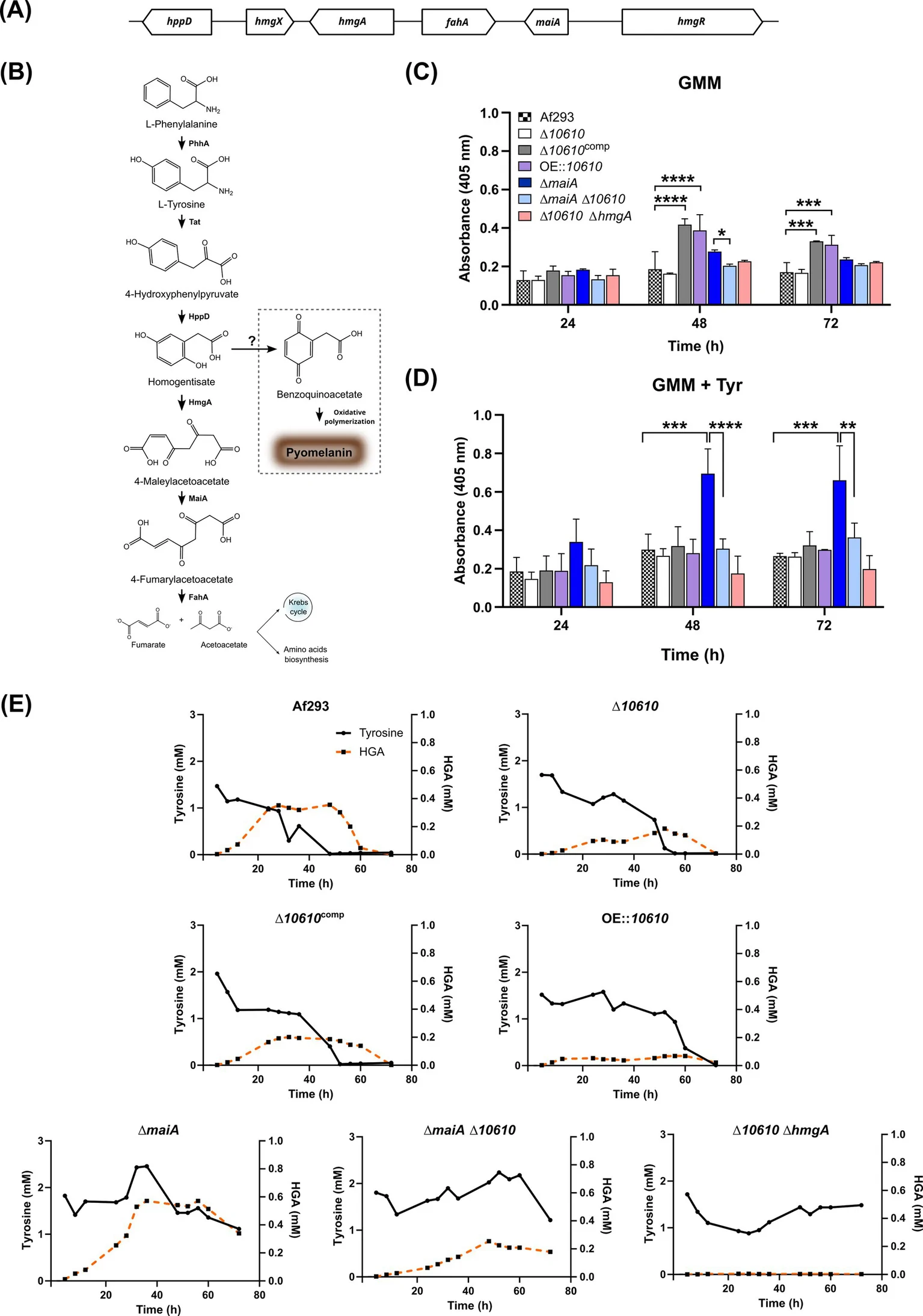
Phenylalanine/tyrosine degradation pathway and pyomelanin production. **(A)** Genetic cluster of phenylalanine/tyrosine degradation pathway. **(B)** Schematic representation of the pathway, including the intermediate metabolites and the enzymes involved. **(C,D)** Quantification of pyomelanin production measured as absorbance at 405 nm in mutant strains over 72 h in **(C)** GMM broth and **(D)** GMM supplemented with 10 mM tyrosine. Statistical analyses were performed using two-way ANOVA followed by Tukey’s multiple comparisons test. Statistical significance is indicated as follows: *p < 0.05, **p < 0.01, ***p < 0.001, ****p < 0.0001. **(E)** Tyrosine (Tyr) consumption and homogentisic acid (HGA) production in different mutant strains. 1 × 10^7^ conidia of each strain were cultivated in 150 mL of GMM broth for 20 h prior to the addition of L-Tyr which was the starting time (time 0) for the measurements. Samples were collected at 12 time points up to 72 h of culture, and Tyr and HGA concentrations were quantified by HPLC.

Pyomelanin biosynthesis in *A. fumigatus* is mediated by a gene cluster involved in phenylalanine and tyrosine catabolism (Figure 4A). This catabolic pathway leads to pyomelanin production through conversion of HGA into BQA (Figure 4B). Although Afu4g10610 is not annotated as a component of this pathway, its predicted association with aromatic amino acid metabolism led us to evaluate a possible role in pyomelanin biosynthesis. To this end, pyomelanin production was quantified by measuring absorbance at 405 nm, in the supernatants of the following strains: Δ*10610*, ∆*10610*^comp^, OE:*10610*, Δ*maiA*, Δ*maiA* Δ*10610*, and Δ*10610* Δ*hmgA*, grown in GMM broth with or without 10 mM tyrosine for 72 h.

In GMM, the ∆*10610*^comp^ and OE:*10610* strains showed increased pyomelanin production compared to the WT strain. This effect was evident under basal conditions, suggesting that increased Afu4g10610 gene expression may enhance pyomelanin production when the pathway is not strongly induced. In contrast, the absence of the gene in the Δ*10610* strain did not appear to affect pyomelanin levels, with the exception of the double mutant Δ*maiA* Δ*10610*, which produced significantly less pigment than Δ*maiA* at 48 h (Figure 4C).

Supplementation with tyrosine induced pyomelanin production in the WT strain, but under these conditions no significant differences were observed between the WT and the ∆*10610*^comp^ and OE:*10610* strains (Figure 4D). In agreement with previous observations (Guruceaga et al., 2024), high pyomelanin production was detected in the Δ*maiA* strain, consistent with its direct involvement in the Phe/Tyr degradation pathway. However, the double mutant Δ*maiA* Δ*10610* exhibited a significant reduction in pyomelanin levels relative to Δ*maiA*, between 48 and 42% at 48 h and 72 h, respectively. Furthermore, the Δ*10610* Δ*hmgA* mutant showed pigment levels similar or even lower than those of the WT strain. Together, these data suggest that Afu4g10610 protein may modulate pyomelanin production.

To determine whether Phe and Tyr affected strain growth, additional qualitative growth assays were performed on solid media using different carbon and nitrogen sources, including GMM, GMM without NaNO₃, and MM (without glucose), supplemented with 10 mM phenylalanine or tyrosine where indicated (Supplementary Figure S3F). Supplementation with Tyr or Phe did not affect the growth of these strains. Only minor qualitative differences were observed, including a more pronounced fluffy colony morphology in the Af293 strain, together with variations in colony pigmentation and sectoring on MM, suggesting limited effects on colonial morphology under these conditions.

HPLC analyses were performed to quantify tyrosine and HGA levels in the different mutant strains growing in GMM broth supplemented with 10 mM of Tyr for 72 h (Figure 4E). Tyrosine was selected as the starting substrate of the phenylalanine/tyrosine degradation pathway, whereas HGA represents a key intermediate and direct precursor of pyomelanin biosynthesis (Figure 4B). Therefore, quantification of these metabolites in culture supernatants allows the assessment of flux through the pathway and potential alterations in pyomelanin production. To integrate metabolite dynamics over time, AUC values were calculated for Tyr (AUC_Tyr_) and HGA (AUC_HGA_) levels throughout the incubation period (Supplementary Table S3). In the wild-type strain, tyrosine consumption correlated with HGA production. Tyrosine was fully consumed after approximately 48 h (AUC_Tyr_: 35.71 mM·h). HGA production increased during the early stages of growth, reaching a peak of 0.35 mM at 24 h, remaining stable until 48 h, and subsequently decreasing (AUC_HGA_: 13.87). In contrast, HGA levels were reduced in the Δ*10610* mutant, with a maximum of 0.18 mM (AUC_HGA_: 6.09), and a similar trend was observed in the ∆*10610*^comp^ strain. The overexpression strain exhibited a delay in tyrosine consumption (AUC_Tyr_: 73.04) and only low levels of HGA were detected (maximum concentration: 0.07 mM; AUC_HGA_: 3.2).

Regarding mutants in key enzymatic steps of the pathway, altered tyrosine consumption and HGA dynamics were observed, consistent with their role in the Phe/Tyr catabolism. Tyrosine consumption and HGA accumulation appear uncoupled, suggesting altered metabolic flux distribution within the pathway. In the Δ*maiA* background, high levels of both tyrosine and HGA were detected (AUC_Tyr_: 114.19; AUC_HGA_: 25.77). In contrast, the double mutant Δ*maiA* Δ*10610* showed markedly lower HGA levels compared to Δ*maiA* (AUC_HGA_: 9.3). Finally, the Δ*10610* Δ*hmgA* strain failed to produce detectable HGA, indicating a strong impairment in this step of the pathway. Overall, the absence of Afu4g10610 in Δ*10610*, Δ*maiA* Δ*10610*, and Δ*10610* Δ*hmgA* strains was associated with markedly reduced HGA levels compared to strains with normal expression, suggesting a role for the Afu4g10610 protein in HGA metabolism.

Considering the connection between HGA metabolism and pyomelanin biosynthesis, the observed alterations in HGA levels point to a functional link between Afu4g10610 protein and this pathway. Taken together with the cell wall and osmotic stress phenotypes, these findings support a broader role for the Afu4g10610 protein in both stress adaptation and aromatic amino acid metabolism in *A. fumigatus*.

### 3.4 Afu4g10610 protein influences host-cell interaction *in vitro*

To evaluate whether Afu4g10610 protein contributes to host-pathogen interactions, and given its previously observed upregulation in both RAW 264.7 murine macrophages and A549 human epithelial cells, we performed infection assays using these *in vitro* systems.

**Figure 5:**
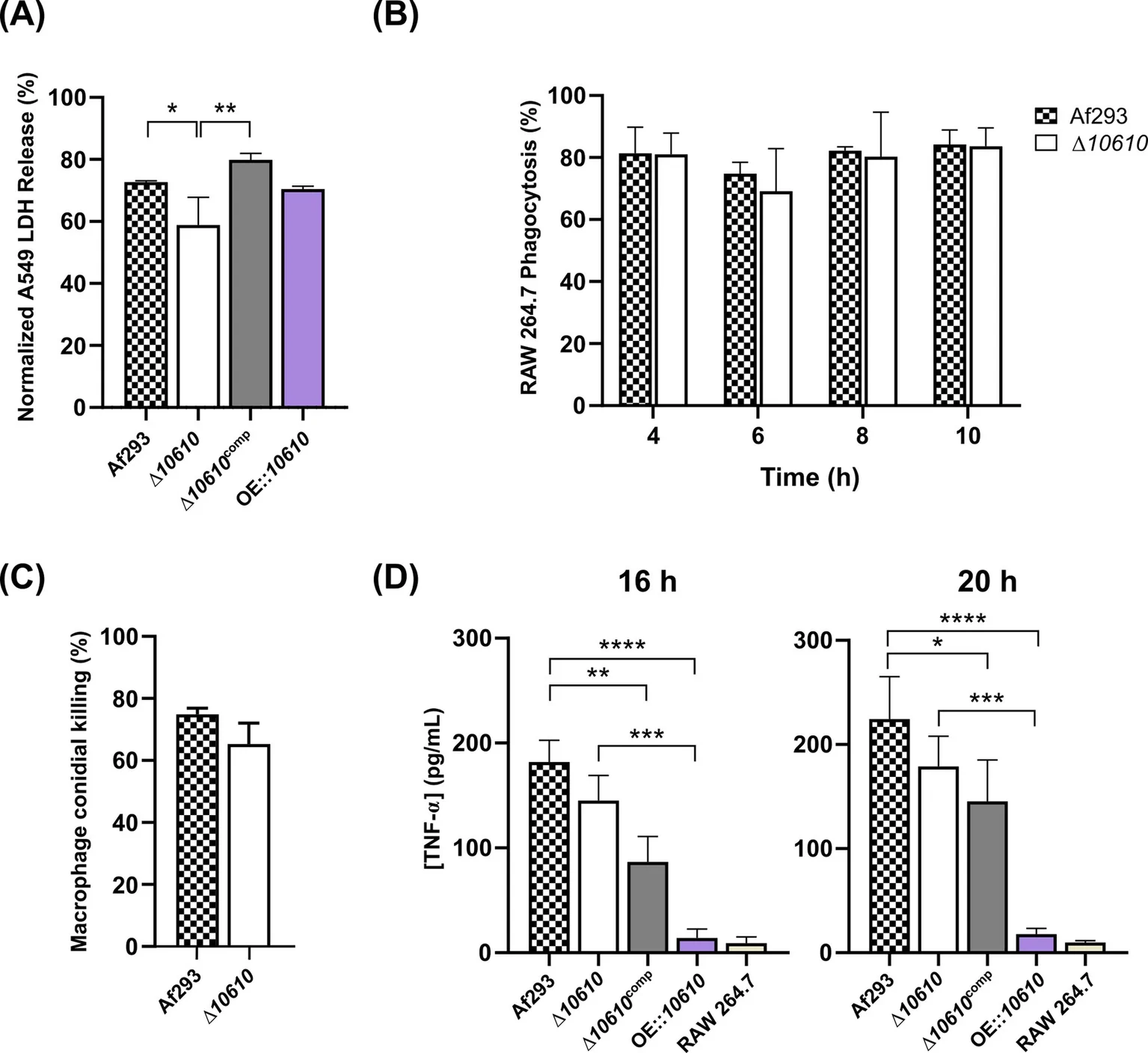
*In vitro* infection assays of *A. fumigatus* strains. **(A)** Cytotoxicity measured as lactate dehydrogenase (LDH) release in A549 human epithelial cells after 16 h of infection (MOI = 1). Data were normalized to positive and negative controls. **(B)** Phagocytosis (%) of Af293 and Δ*10610* conidia by RAW 264.7 murine macrophages during macrophage interaction. **(C)** Macrophage-mediated killing of conidia, expressed as the percentage of phagocytosed conidia killed relative to the initial inoculum after 6 h of interaction (MOI = 2). **(D)** TNF release by RAW 264.7 macrophages quantified by ELISA after 16 h and 20 h of infection (MOI = 1), following the manufacturer’s instructions. Data represent the mean ± SD of three independent experiments. Statistical analyses were performed using two-way ANOVA followed by Tukey’s multiple comparisons test. Statistical significance is indicated as follows: *p < 0.05, **p < 0.01, ***p < 0.001, ****p < 0.0001.

Epithelial cytotoxicity was quantified by measuring LDH release in A549 culture supernatants after 16 h of infection at a multiplicity of infection (MOI) of 1. Cultures infected with the Δ*10610* strain resulted in significantly reduced LDH release compared to Af293 strain, suggesting a decreased cytotoxicity (Figure 5A). In contrast, the complemented and overexpression strains induced LDH levels comparable to those of WT strain.

On the other hand, we assessed the phagocytosis and phagocytic killing of conidia using RAW 264.7 macrophages. No differences were observed in the phagocytosis percentage of the Δ*10610* mutant compared to the WT strain during interaction with macrophages (MOI = 1) (Figure 5B). Similarly, no significant differences in macrophage killing of conidia were observed among the strains tested after 6 h of interaction (MOI = 2) (Figure 5C). Furthermore, TNF, IL-6 and IL-1*β* cytokine secretion by macrophages was quantified by ELISA after 16 and 20 h of infection (MOI = 1). Regarding TNF, a slight decrease in production was observed in the Δ*10610* mutant compared to the WT strain. However, a marked reduction in TNF production was observed in response to the ∆*10610*^comp^ and OE:*10610* strains, compared to the wild type (Figure 5D), suggesting a diminished pro-inflammatory stimulation by these strains. In addition, the other cytokines assessed, including IL-6 and IL-1β, were not detected under the conditions tested (data not shown). Overall, these results suggest that the Afu4g10610 protein may contribute to fungal virulence during host-cell interactions.

### 3.5 Proposed functional model of Afu4g10610 protein

**Figure 6:**
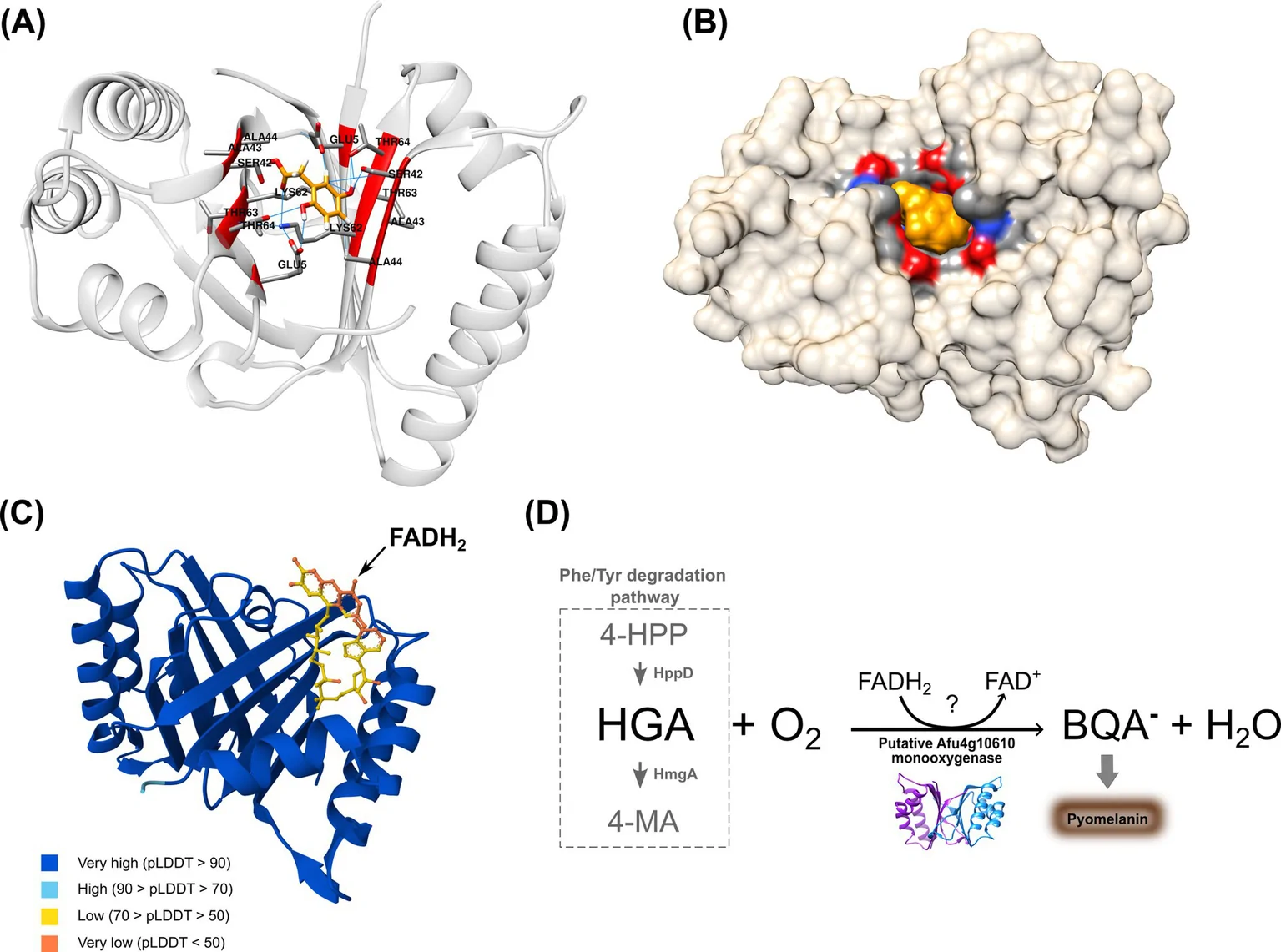
Proposed functional model of Afu4g10610 protein by bioinformatic analysis: **(A, B)** Docking of homogentisic acid (HGA) in the dimeric Afu4g10610 pocket predicted by SwissDock. **(A)** Cartoon representation of the dimer, highlighting the overall fold and the most probable orientation of HGA within the pocket. Dimeric Afu4g10610 protein is shown in white, HGA in orange, amino acids involved in substrate binding are colored red, and hydrogen bonds are indicated in blue. Additional interactions are not shown for clarity. These include a cation-*π* interaction between the aromatic ring of HGA and LYS62, ionic contacts (GLU5-LYS62, LYS62-HGA(O_2_) per monomer), and hydrophobic contacts (HGA(C6)-ALA44, HGA(C6)-LYS62, ALA44-GLU5, GLU5-LYS62 per monomer). **(B)** Surface representation of the docking, showing the topography of the protein and the pocket where HGA fits snugly within the dimer interface. **(C)** AlphaFold3 predictive model of the FADH_2_-Afu4g10610 dimer interaction. **(D)** Proposed reaction for the oxygenation of HGA into benzoquinone acetate (BQA), catalyzed by the putative flavin-dependent monooxygenase Afu4g10610. 4-HPP: 4-hydroxyphenylpyruvate; HGA: homogentisic acid; BQA: benzoquinone acetate; 4-MA: 4-maleylacetoacetate.

In *A. fumigatus*, pyomelanin biosynthesis is preceded by the conversion of HGA into BQA, a reaction involving monooxygenation of the aromatic ring (Figure 4B). Due to its high reactivity, BQA undergoes a rapid polymerization, leading to pyomelanin formation. The Afu4g10610 protein is predicted to form a homodimer adopting an *α*/β-barrel architecture, typical structure of enzymes that display monooxygenase activity. Two lines of evidence support a role for the Afu4g10610 protein in the conversion of HGA into BQA and, subsequently, in pyomelanin production. On the one hand, overexpression of Afu4g10610 gene in the ∆*10610*^comp^ and OE:*10610* strains was associated with increased pyomelanin production compared to the WT strain. On the other hand, double mutant lacking this protein (∆*maiA* ∆*10610*) showed reduced pigment production and lower HGA levels compared to the hyperpigmented parental strain ∆*maiA*. A similar trend was observed in the ∆*10610* ∆*hmgA* strain. These results suggest that the HGA-to-BQA conversion step could represent a key point influencing pyomelanin biosynthesis. Based on these observations, we hypothesized that Afu4g10610 could contribute to this process, either directly or through interactions with other pathway components. To support this hypothesis, we performed molecular docking of HGA into the protein using the bioinformatics tool SwissDock, which employs the Attracting Cavities and AutoVina methods for its analyses. Surprisingly, both methods positioned HGA at the dimer interface, with docking results consistent with a transient substrate binding, typical of catalytic processes (Figures 6A,B). (Attracting Cavities: AC Score: −40.7 and SwissParam Score: −6.6; AutoVina: Calculated affinity: −5.269 kcal·mol^−1^; Supplementary Figure S2D). This pose is stabilized by multiple hydrogen bonds and ionic interactions with amino acids GLU5, SER42, ALA43-44, THR63-64, and LYS62, which interact across both monomers (Figure 6A). In addition, other types of interactions are also present, including a cation-*π* interaction between the aromatic ring of HGA and LYS62, ionic contacts (GLU5-LYS62, LYS62-HGA(O_2_) per monomer), and hydrophobic contacts (HGA(C6)-ALA44, HGA(C6)-LYS62, ALA44-GLU5, GLU5-LYS62 per monomer).

Furthermore, docking analyses using AlphaFold3 and SwissDock predicted favorable binding of the flavin adenine dinucleotide (FADH₂) at the dimer interface. The dimer model predicted by AlphaFold yielded a pTM score of 0.88 and an ipTM score of 0.84 (Figure 6C), exceeding established thresholds (pTM > 0.5 and ipTM > 0.8), indicating a reliable overall fold and a high-confidence prediction of subunit positioning within the complex (Supplementary Figure S2C). In parallel, SwissDock analysis resulted in multiple distinct energetically favorable poses (Attracting cavities: AC Score = −183.4; SwissParam Score = −9.0; AutoVina: −5.3 kcal·mol^−1^) (Supplementary Figure S2E). Therefore, while these data strongly suggest that the protein can also accommodate a flavin cofactor, experimental validation is required to confirm FAD dependence and to identify the FAD-binding site.

The fit of HGA within the dimer interface, the ability of the protein to accommodate FADH₂ as a potential electron donor, and the phenotypic changes observed in mutants of enzymes involved in this pathway, support a model in which Afu4g10610 may function as a flavin-dependent monooxygenase involved in the conversion of HGA to BQA, at least in part (Figure 6D). Nonetheless, given that the ∆*10610* mutant still produces detectable levels of pyomelanin, we cannot exclude spontaneous BQA formation or the involvement of additional monooxygenases. Importantly, pyomelanin metabolism is tightly linked to cell wall homeostasis, and therefore this model is consistent with the dysregulated osmotic and cell wall stress responses observed in the Afu4g10610 mutants.

## 4 Discussion

The biology of the pathogenic fungus *A. fumigatus* remains incompletely understood, in part due to the large number of hypothetical and uncharacterized proteins. In this study, we focused on Afu4g10610, a protein of unknown function that emerged from multiple stress-associated transcriptomic datasets, including both *in vitro* and *in vivo* infection models (Guruceaga et al., 2024). The phenotypic analyses presented here suggest that its contribution becomes most apparent under conditions that challenge fungal adaptation rather than during basal growth. Therefore, we investigated the function of Afu4g10610 protein through the generation of mutant strains. The ∆*10610* strain exhibited an impaired capacity to respond to cell wall stressors such as CR and CFW. This phenotype correlates with the reduced expression of the cell wall stress sensors *wsc1* and *midA* (Dichtl et al., 2012), key components of the CWI pathway, suggesting that Afu4g10610 may influence the cellular response to cell wall stress. However, no differences were observed in cell wall architecture by scanning electron microscopy or in the expression of genes involved in cell wall biosynthesis and remodeling, including *cwh41*, *fks1*, *chsA*, and *chsC*, between the WT strain and the deletion mutant. This is consistent with the lack of effect observed for antifungal agents targeting the cell wall, including echinocandins and nikkomycin Z. Together, these results suggest that Afu4g10610 contributes to the adaptation to cell wall stress rather than to basal cell wall architecture.

The ∆*10610* strain also exhibited altered responses to osmotic stress, showing increased resistance, accompanied by significant upregulation of *mpkC*. A slight, although not statistically significant, increase in *sakA* expression was also observed. Both *mpkC* and *sakA* have been described as interacting components of the HOG pathway involved in fungal adaptation to osmotic and cell wall stress conditions (Manfiolli et al., 2019). In addition, the ∆*10610,* ∆*10610*^comp^ and OE:*10610* strains also showed increased growth under osmotic stress conditions, and sorbitol supplementation improved growth under CR- and CFW-induced cell wall stress, reaching levels higher than those observed in the WT strain. These phenotypes are consistent with the protective effect of osmotic stabilizers on cell wall integrity (Lamoth et al., 2012; Richie et al., 2009). Although further studies are required to determine whether these changes are causative or consequential, the data suggest that Afu4g10610 expression level may influence adaptation to both cell wall and osmotic stress. Supporting this notion, the ∆*10610*^comp^ and OE:*10610* strains partially restored the stress-response phenotype, but did not fully phenocopy the wild-type strain, which suggests that the phenotype may be sensitive to Afu4g10610 expression level or genomic context. Importantly, multiple independent complementation strategies were tested in this study, all of which consistently resulted in only partial phenotypic rescue. One possible explanation is that expression from the complemented locus does not fully recapitulate the native regulatory context. Overall, these findings suggest that Afu4g10610 is not required for basal cell wall architecture, but rather contributes to adaptive responses to cell wall and osmotic stress.

Furthermore, using GRAsp (Carriel et al., 2026), we identified potential relationships between the Afu4g10610 gene and (i) genes involved in the Phe/Tyr degradation pathway, which is linked to the production of the secondary metabolite pyomelanin, (ii) the monooxygenase encoded by Afu6g02210, and (iii) the indoleamine 2,3-dioxygenase (*idoC*). Notably, several of these genes, including Afu1g04160, *hppD*, *maiA*, Afu6g02210, and *idoC*, were found to be overexpressed together with Afu4g10610 under the stress conditions analyzed in our previous study (Guruceaga et al., 2024). GRAsp has also been used to predict functional relationships in *A. fumigatus*, such as the roles of the RogA and HsfA TFs in gliotoxin regulation (Seo et al., 2025) or the bZIP TF AtfA in fungal responses to microbial signals (Carriel et al., 2026). In this context, GRAsp provided a useful framework for prioritizing candidate functional links for experimental evaluation. These associations should nevertheless be interpreted considering that *A. fumigatus* contains homologous enzymes with similar predicted activities that may function in distinct biological contexts. For example, Afu4g10620 gene, located upstream of Afu4g10610, encodes a predicted 4-hydroxyphenylpyruvate dioxygenase, as does *hppD* (Afu2g04200), involved in the Phe/Tyr degradation pathway. Therefore, although GRAsp provides valuable hypotheses for functional associations, these relationships should be interpreted within the broader biological context of *A. fumigatus.* The phenylalanine/tyrosine degradation pathway has been extensively studied because of its relevance to *A. fumigatus* biology and its direct link to the production of the secondary metabolite pyomelanin (Keller et al., 2011; Schmaler-Ripcke et al., 2009). To assess the potential involvement of Afu4g10610 in this pathway, pyomelanin production was measured in different mutant strains. In GMM, the Δ*10610* strain did not show a significant reduction in pyomelanin production relative to the wild-type strain, whereas the Δ*10610*^comp^ and OE:*10610* strains produced higher levels of this metabolite. Although increased gene expression does not necessarily imply a functional effect, the elevated expression of Afu4g10610 in these strains may correlate with enhanced pyomelanin production. At the same time, the persistence of basal pyomelanin levels in the Δ*10610* mutant, together with unchanged *hmgA* and *hppD* expression, indicates that Afu4g10610 is unlikely to be the sole determinant of pyomelanin formation.

When tyrosine was added to force the pathway, mutants in key enzymes of phenylalanine/tyrosine catabolism, such as Δ*maiA* and Δ*hmgA*, have been reported to accumulate high levels of pyomelanin (Guruceaga et al., 2024; Heinekamp et al., 2013; Schmaler-Ripcke et al., 2009). Consistent with these previous observations, the Δ*maiA* mutant also produced high levels of pyomelanin in this study. In contrast, the Δ*maiA* Δ*10610* strain showed a marked reduction in pyomelanin compared with its hyperpigmented parental Δ*maiA* strain, together with lower tyrosine consumption and reduced HGA production. A similar effect was observed in the Δ*10610* Δ*hmgA* mutant, which produced even less pyomelanin than the wild type and showed undetectable HGA levels. However, as the hyperpigmented Δ*hmgA* strain could not be obtained despite repeated attempts, the same genetic comparison performed between the Δ*maiA* and Δ*maiA* Δ*10610* strains could not be established for the Δ*10610* Δ*hmgA* background. Although this limitation prevents a direct interpretation of the Δ*10610* Δ*hmgA* phenotype, the reduction in pyomelanin production in both double mutants is consistent with a contribution of Afu4g10610 to pyomelanin formation. Notably, unlike the single Afu4g10610 mutant, these pathway-enzyme mutants also showed sustained tyrosine levels over time, suggesting broader disruption of pathway coordination. One possible explanation is that, in the absence of Afu4g10610, HGA metabolism is altered, resulting in reduced flux toward pyomelanin formation and a possible redistribution through alternative detoxification routes, particularly in backgrounds where pathway flux is already perturbed. This effect may be more pronounced in the Δ*10610* Δ*hmgA* mutant, where downstream metabolism is further constrained. Together, these findings support a context-dependent contribution of Afu4g10610 to pyomelanin formation, particularly under conditions of increased pathway flux or genetic perturbation.

Interestingly, previous studies have also reported a connection between the CWI pathway and pyomelanin production. Valiante et al. (2009) report that under normal conditions, the MpkA module regulates HmgA and HppD activity at the post-transcriptional level, resulting in reduced HmgA activity. Upon cell wall stress, these authors also reported that MpkA becomes hyperphosphorylated, inducing an imbalance between HmgA and HppD that leads to increased pyomelanin production. Based on this model, we hypothesize that in the ∆*10610* strain, this stress-induced increase in pyomelanin may be impaired, raising the possibility that an impaired pyomelanin response under cell wall stress could contribute to the observed phenotype. Future experiments could assess the ability of the mutant strains to produce pyomelanin under cell wall stress conditions. Another experiment that could provide further insight into the Afu4g10610 function would be the use of phenylalanine as substrate. However, phenylalanine was not used in this study because tyrosine is thought to force the degradation pathway more efficiently, while phenylalanine can be more readily utilized as an alternative carbon source (Guruceaga et al., 2024).

Taken together, the genetic and metabolic data suggest that Afu4g10610 may contribute to the HGA-to-BQA conversion step during pyomelanin biosynthesis. This possibility is further supported by the predicted structural features of the protein. Afu4g10610 is predicted to adopt a dimeric *α*/*β*-barrel architecture, a fold commonly associated with monooxygenase activity (Revington et al., 2005; Sciara, 2003), although experimental validation of its oligomeric state is still required. Given that the conversion of HGA to BQA involves monooxygenation and represents a key step in pyomelanin biosynthesis, we investigated the potential interaction between Afu4g10610 and HGA using bioinformatic approaches. Molecular docking analyses using SwissDock positioned HGA at the predicted dimer interface. This pose was stabilized by multiple hydrogen bonds and ionic interactions involving residues GLU5, SER42, ALA43-44, THR63-64, and LYS62, which contribute to interactions between both monomers (Figure 6A). In addition, favorable binding of the cofactor FADH₂ was also predicted, suggesting that FADH₂ could potentially serve as a redox cofactor compatible with a monooxygenase-like mechanism. Future experimental validation of this model will require biochemical characterization of recombinant Afu4g10610, including site-directed mutagenesis of residues predicted to participate in the HGA binding and assessment of their impact on substrate interaction and enzymatic activity. Together with the altered HGA levels observed in mutants lacking Afu4g10610, these findings support Afu4g10610 as a plausible candidate contributing to HGA binding and oxidation during pyomelanin biosynthesis. Although our data support a mechanistic model linking Afu4g10610 to HGA metabolism, they do not exclude additional roles in fungal physiology and stress adaptation. Indeed, the functional diversity reported for proteins adopting Dabb architectures in both other organisms and *A. fumigatus* indicates that structural similarity alone is insufficient to define biological function. Therefore, further studies will be required to determine whether Afu4g10610 contributes more broadly to *A. fumigatus* physiology beyond its proposed role in pyomelanin metabolism.

The Phenylalanine/tyrosine degradation pathway has also been studied due to its role in virulence (Heinekamp et al., 2013; Keller et al., 2011). Although *hmgR* and *hppD* have not been linked to the pathogenicity of *A. fumigatus*, the *∆maiA* mutant exhibits significantly reduced virulence compared to the wild-type strain in a chemotherapeutically immunosuppressed murine infection model (Guruceaga et al., 2024). In this project, the potential contribution of Afu4g10610 to host-cell interactions was assessed using *in vitro* models. The ∆*10610* mutant showed a significantly reduced cytotoxicity in pulmonary epithelial A549 cells, but only a slight reduction in TNF production by RAW 264.7 macrophages. In contrast, macrophages challenged with the ∆*10610*^comp^ and OE:*10610* strains produced considerably lower levels of TNF. Both strains also exhibited increased pyomelanin production under normal conditions and elevated expression of the Afu4g10610 gene. Although the role of pyomelanin in virulence remains unclear, these observations are consistent with the hypothesis that increased pyomelanin production may have an immunomodulatory effect on macrophages (Heinekamp et al., 2013). Interestingly, the different TNF responses observed among the ∆*10610*, ∆*10610*^comp^, and OE:*10610* strains suggest that the effect of Afu4g10610 on macrophage activation is not directly proportional to gene expression levels. Although ∆*10610*^comp^ and OE:*10610* strains showed increased Afu4g10610 expression and pyomelanin production, they did not simply restore a wild-type phenotype, suggesting that altered Afu4g10610 levels may influence the host response in a more complex manner. Other inflammatory cytokines, such as IL-6 and IL-1β, were not detected under the tested conditions. Further studies incorporating complementary approaches will be necessary to elucidate the role of this protein in host-pathogen interactions. These approaches may include analyses of epithelial integrity, host signaling activation, and host-pathogen interaction markers (Okaa et al., 2023), as well as additional infection parameters (e.g., higher MOI, alternative macrophage models, or extended stimulation times) (Li et al., 2026; Pinzan et al., 2025). Moreover, given that Afu4g10610 gene was initially identified from *in vivo* infection transcriptomic datasets, future studies using animal infection models will be required to determine its contribution to fungal virulence.

In conclusion, our data suggest that HGA-to-BQA conversion in *A. fumigatus* may not rely exclusively on spontaneous oxidation. Instead, Afu4g10610 emerges as a stress-associated protein that links adaptive stress responses with pyomelanin metabolism in *A. fumigatus*. The combined genetic, metabolic, and docking results are consistent with a possible flavin-associated role, but direct biochemical validation will be required to establish substrate specificity, catalytic activity, and cofactor dependence. To our knowledge, this study provides the first evidence in *A. fumigatus* pointing to the possible involvement of a flavin-associated protein in this step of pyomelanin biosynthesis.

## Data Availability

The original contributions presented in the study are included in the article/Supplementary material, further inquiries can be directed to the corresponding authors.
