# Supplementary material for: A stress responsive protein contributes to pyomelanin biosynthesis and cell wall stress adaptation in Aspergillus fumigatus
Source: Front Microbiol. 2026 Sep 3;17:1934017. doi: 10.3389/fmicb.2026.1934017 (PMC13582290; doi:10.3389/fmicb.2026.1934017)
Supplement: Supplementary file 1 [file Data_Sheet_1.docx]

Supplementary Material

**Figure S1. Schematic representation of CRISPR/Cas9-mediated gene editing strategies. (A)** Deletion of Afu4g10610 was carried out using two guide RNAs (gRNA) targeting sequences flanking the gene. A hygromycin resistance cassette (HygR) containing 40 bp microhomology regions served as repair template. **(B)** Complementation of the ∆*10610* mutant was performed by fusing the native Afu4g10610 gene to a phleomycin resistance cassette (PhleoR) through fusion PCR. Two gRNAs targeting upstream and downstream regions of the ∆*10610* locus directed homologous recombination with the repair cassette. **(C)** Overexpression strategy. A single gRNA targeting the 5′ region of Afu4g10610 was designed. The double-strand break was repaired using a cassette containing the *hspA* promoter upstream of the gene and a PhleoR downstream. **(D)** Double deletion mutants. For example, in the ∆*maiA* background, the ∆*10610* deletion was generated using a PhleoR as a repair template. Other double mutants were constructed similarly. All transformants were confirmed by PCR and sequencing.

**Figure S2. Bioinformatic analysis of Af4g10610 protein. (A-C)** AlphaFold-predicted models of the Afu4g10610 protein: **(A)** monomer, **(B)** dimer, and **(C)** dimer in complex with FADH₂. Models show high predicted local distance difference test (pLDDT) scores and low predicted aligned error (PAE), indicating high confidence in both local residue positioning and overall structure. **(D)** List of the most probable poses obtained from molecular docking of HGA to the dimeric Afu4g10610 protein using SwissDock. Poses were ranked according to the Attracting Cavities method (AC Score and SwissParam Score) and AutoVina (calculated affinity in kcal·mol^-1^). **(E)** List of the most favorable FADH₂ docking poses in the dimeric Afu4g10610 protein, obtained using the Attracting Cavities and AutoVina methods.

**Figure S3.** **Phenotypic characterization of Af293 and ∆*10610*.** **A, B.** Growth on GMM 6-well plates supplemented with increasing concentrations (0, 0.5, 1, 2, 4, and 8 μg/mL) of nikkomycin Z **(A)** or caspofungin **(B)**. Plates were inoculated with 1 × 10⁴ fresh conidia of each strain and incubated at 37°C for 72 h. **(C)** Drug strip diffusion assay for the assessment of echinocandin and azole susceptibility. 1 × 10⁶ conidia of the indicated strains were spread onto GMM agar plates. After drying, strips containing caspofungin, micafungin, voriconazole, or posaconazole were applied, and plates were incubated at 37°C for 48 h. **(D)** Scanning electron microscopy of hyphae from strains grown in GMM broth for 16 h. **(E)** Growth of strains on GMM supplemented with 1.2 M NaCl or KCl. Plates were incubated at 37°C, and colony diameters were measured at 24, 48, and 72 h. Data were analyzed by one-way ANOVA (n = 3; mean ± SD). Statistical significance is indicated as follows: **p* < 0.05, ***p* < 0.01. **(F, G)** Growth assays under different nutritional conditions. Strains were spotted (1 × 10⁴, 10³, and 10² conidia) onto **(F)** GMM, GMM supplemented with 10 mM tyrosine, GMM without NaNO₃ supplemented with 10 mM tyrosine (tyrosine as the sole nitrogen source), minimal medium (MM) lacking glucose and NaNO₃ supplemented with 10 mM tyrosine (tyrosine as the sole carbon and nitrogen source), and MM supplemented with 10 mM phenylalanine. **(G)** GMM incubated at 48°C, GMM supplemented with 1.2 M sorbitol and incubated at 48°C, GMM supplemented with 5 mM caffeine, and GMM adjusted to pH 9. Plates were incubated for 72 h.

**Table S2**. HPLC-PDA conditions for the analysis of tyrosine and HGA in GMM culture media

| Parameter | Conditions |
| --- | --- |
| Column | C18 Zorbax Eclipse XDB (15 cm × 4.6 mm, 5 µm) |
| Column Temperature (^o^ C) | 35 |
| Mobile phase | Aqueous phase “A” (H_2_O, 0,01% TFA)  Organic phase “B” (100% ACN) |
| Elution mode | 10 % B for 4 min, gradient from 10 % B to 100 % B within 4 min, 100 % B for 2 min, gradient to 100% B to 10 % B within 3 min and, finally, 5 min at 10 % B for recalibration. |
| Flow rate (mL/min) | 1 |
| Injection volume (µL) | 10 |
| Autosampler temperature (^o^ C) | 4 |
| Analysis time (min) | 18 |
| Wavelength (nm) | 270 nm (tyrosine)  290 nm (HGA) |

**Table S3**. Area under the curve analysis of tyrosine and homogentisic acid levels during the 72 h
time-course

| Strain | | AUC_Tyr_ (mM∙h) | AUC_HGA_ (mM∙h) | Max [HGA] (mM) | Time of Max [HGA] | |
| --- | --- | --- | --- | --- | --- | --- |
| Af293 | 35.71 | | 13.87 | 0.35 | 48 h |  |
| ∆*10610* | 55.23 | | 6.09 | 0.18 | 52 h |  |
| ∆*10610*^comp^ | 50.97 | | 8.78 | 0.20 | 32 h |  |
| OE::*10610* | 73.04 | | 3.20 | 0.07 | 60 h |  |
| ∆*maiA* | 114.19 | | 25.77 | 0.57 | 56 h |  |
| ∆*maiA* ∆*10610* | 120.17 | | 9.30 | 0.25 | 48 h |  |
| ∆*10610* ∆*hmgA* | 84.12 | | 0.19 | 0.00 | 28 h |  |

AUC: Area Under the Curve; Tyr: Tyrosine; HGA: Homogentisic acid
